# Epirubicin induces cardiotoxicity through disrupting ATP6V0A2-dependent lysosomal acidification and triggering ferroptosis in cardiomyocytes

**DOI:** 10.1038/s41420-024-02095-z

**Published:** 2024-07-24

**Authors:** Mingming Zhang, Xin Wu, Yuting Wen, Zhiquan Li, Fuzhong Chen, Yu Zou, Xiaoyu Dong, Xinjian Liu, Junhong Wang

**Affiliations:** 1 Department of Cardiology, the first affiliated hospital of Nanjing Medical University, 300 Guangzhou Road, Nanjing, China; 2 Department of Obstetrics, the first affiliated hospital of Nanjing Medical University, 300 Guangzhou Road, Nanjing, China; 3 Department of Pathogen Biology, Nanjing Medical University, 101 Longmian Avenue, Nanjing, China; 4 Key Laboratory of Antibody Technique of National Health Commission of China, Nanjing Medical University, 101 Longmian Avenue, Nanjing, China

**Keywords:** Cardiovascular diseases, Experimental models of disease

## Abstract

Epirubicin (EPI) is effective in the treatment of malignant cancers, but its application is limited by life-threatening cardiotoxicity. Iron homeostasis disturbance has been implicated in anthracycline induced cardiotoxicity (AIC), and ferroptosis is involved in AIC which dependent upon intracellular iron. However, the role and exact mechanisms of ferroptosis in the pathogenesis of epirubicin-induced cardiotoxicity (EIC) remain elusive. In this study, we aimed to investigate mechanisms underlying ferroptosis-driven EIC. Epirubicin triggered ferroptosis both in vivo and in cultured cardiomyocytes, and pretreatment with ferroptosis inhibitor, Ferrostatin-1(Fer-1) alleviates EIC. Microarray analysis was performed to screen for potential molecules involved in EIC in neonatal primary mouse ventricular cardiomyocytes (NMVMs). We found that the transcript level of ATP6V0A2, a subunit of vacuolar ATPase (V-ATPase), was significantly downregulated when NMVMs were subjected to EPI, which was verified in vivo and in vitro as measured by real time quantitative reverse transcription PCR (qRT-PCR) and immunoblotting. Intriguingly, overexpression of ATP6V0A2 effectively decreased excessive oxidative stress and lipid-peroxidation accumulation, thereby inhibiting ferroptosis and protecting cardiomyocytes against EIC, as evidenced by functional, enzymatic, and morphological changes. Mechanistically, forced expression of ATP6V0A2 restored lysosomal acidification in EPI-treated cardiomyocytes and protected cardiomyocytes and mice hearts from ferroptosis-driven EIC. In this study, our data elucidate that ferroptosis is involved in EIC, which is ignited by ATP6V0A2-dependent lysosomal acidification dysfunction. Our study provides a new potential therapeutic target for ameliorating EIC.

## Introduction

Epirubicin (EPI), an efficacious anthracycline, is commonly used in chemotherapeutic. However, its clinical use is limited by dose-dependent cardiotoxicity [1, 2]. The death of terminally differentiated cardiomyocytes plays a vital role in the process of cardiac injury. Current therapies for protecting the heart from EPI induced cardiotoxicity (EIC) are unsatisfactory. Therefore, exploring the specific mechanisms and identifying novel targets to defend against EIC is a high priority.

Apoptosis has been considered the main form of myocardial cell death [3]. Other types of cell death, including autophagy [4, 5], necroptosis [6], and pyroptosis [7, 8], have also been identified to involve in anthracyclines induced cardiotoxicity. Nevertheless, there is accumulating evidence that ferroptosis contributes cardiomyocyte death in various heart diseases, including heart failure (HF) [9], ischemic heart disease [10, 11], myocardial infarction [12, 13] and cardiomyopathy [14, 15] among others. Notably, ferroptosis is a novel form of regulated cell death, characterized by an iron-dependent increase in lipid peroxidation and damage to biological membrane structures [16, 17]. With excessive production of ferrous iron, mounts of reactive oxygen species (ROS) through Fenton reaction were generated [18] so that the lipid hydroperoxides abnormally accumulate on various lipid membranes in cells, resulting in membrane destruction and irreversible cell death [16]. As a significant cell death mechanism, ferroptosis is regulated by specific pathways and is involved in diverse biological contexts. However, the complicated regulation mechanisms of ferroptosis, and how it is integrated into other biological processes are still unclear.

Herein, we identified that ATP6V0A2, a subunit of vacuolar ATPase (V-ATPase), was significantly decreased in EIC. We first verify the role of ATP6VA0A2 in cardiovascular diseases, which considerably alleviates ferroptosis and EIC. Mechanistically, ATP6V0A2 maintains lysosomal acidification to suppresses ferroptosis against EIC. These findings highlight the functional involvement of ATP6V0A2 in lysosomal acidity which modulates ferroptosis and provide a new target towards EIC.

## Results

### ATP6V0A2 is downregulated in EIC

**Fig. 1:**
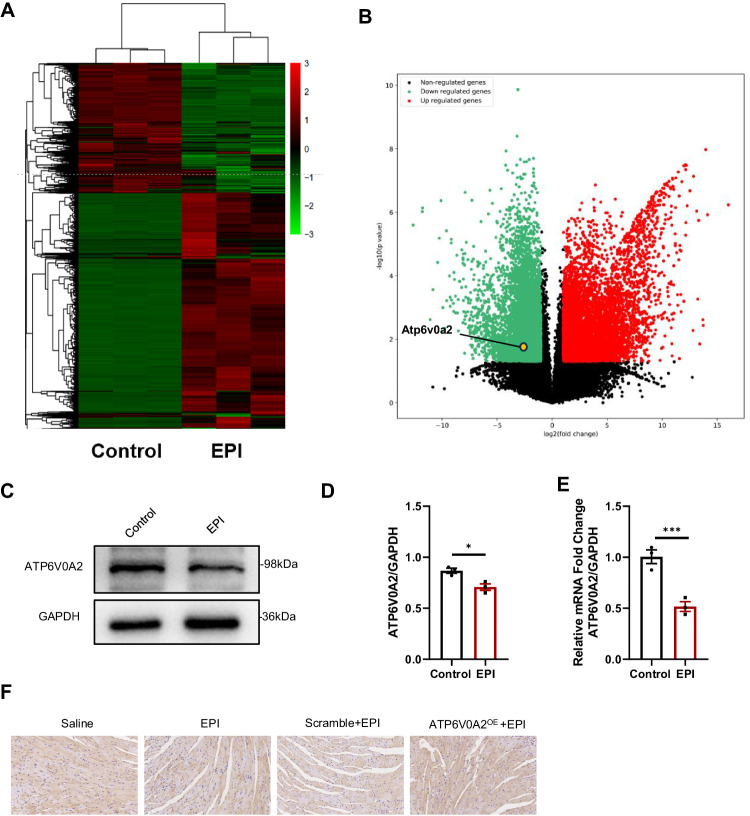
ATP6V0A2 is downregulated in EIC. **A** Heat map showing differentially expressed genes in NMVMs between control and EPI-treated groups. Low expression is depicted in green, and high expression is depicted in red. **B** Volcano plot showing the up-regulated and down-regulated genes in response to EPI treatment measured using microarray analysis. Solid symbols indicate genes in which the differential expression was statistically significant. The orange dot represented ATP6V0A2. **C**, **D** Immunoblotting images of ATP6V0A2 protein in HL-1 cardiomyocytes treated with DMSO or EPI (2 μM) for 12 h. **E** The mRNA level of ATP6V0A2 in HL-1 cardiomyocytes treated with EPI (2 μM). **F** IHC analysis of ATP6V0A2 expression in murine myocardium administrated with DMSO or EPI. Student’s *t* test was performed for statistical analysis. ***p < 0.001, *p < 0.05.

Due to the elusive mechanisms underlying EIC, cDNA microarray analysis was performed to determine differentially expressed genes in NMVMs with or without EPI insult. The heat map and volcano plot reveal that 5621 genes were significantly up-regulated, while 5118 genes were down-regulated in EPI treated NMVMs (Fig. 1A, B). Among these genes, we noticed that ATP6V0A2 is one of the significantly decreased genes. To confirm the reduction of ATP6V0A2, we conducted qRT-PCR and immunoblotting analysis. The results showed that EPI treatment led to a significant decrease in mRNA and protein levels of ATP6V0A2 in cardiomyocytes (Fig. 1C–E). Additionally, histology analysis demonstrated that decreased ATP6V0A2 expression in the hearts of EPI-treated mice (Fig. 1F). Taken together, the findings indicated that ATP6V0A2 was conceivably downregulated during the progression of EIC.

### ATP6V0A2 overexpression alleviates EIC in vitro and in vivo

**Fig. 2:**
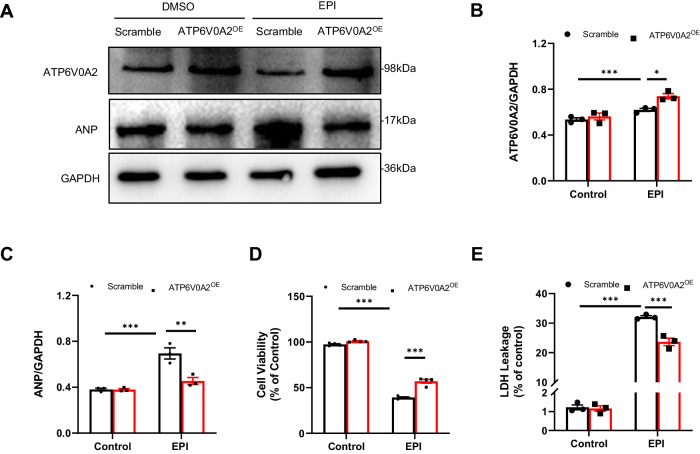
ATP6V0A2 overexpression alleviates EIC in vitro. HL-1 cardiomyocytes were transfected with plasmids of scramble or ATP6V0A2 for 48 h, and then treated with DMSO or EPI (2 μM) for 12 h. **A**–**C** Immunoblotting images of ATP6V0A2 and ANP proteins in HL-1 cardiomyocytes in the indicated groups. **D** Cell viability was detected in HL-1 cardiomyocytes treated as indicated. **E** LDH release in the medium of HL-1 cardiomyocytes in the indicated groups was detected. Two-way ANOVA and subsequent Tukey tests were performed for statistical analysis. ***p < 0.001, **p < 0.01, *p < 0.05.

To evaluate the role of ATP6V0A2 in EIC, various experiments were performed in vitro. Compared to cells transfected with control plasmids, the protein level of ATP6V0A2 in EPI-treated HL-1 cardiomyocytes transfected with ATP6V0A2 increased significantly (Fig. 2A, B). Notably, cell viability markedly decreased in EPI-treated cardiomyocytes, but ATP6V0A2 overexpression mitigated this cell death (Fig. 2D). Consistently, ATP6V0A2 overexpression reduced LDH release in EPI-treated HL-1 cardiomyocytes (Fig. 2E). Additionally, increased ANP protein levels were observed in EPI-treated HL-1 cardiomyocytes, which were significantly restored by ATP6V0A2 overexpression (Fig. 2A, C). Collectively, these results suggested that ATP6V0A2 protected cardiomyocytes from EIC.

**Fig. 3:**
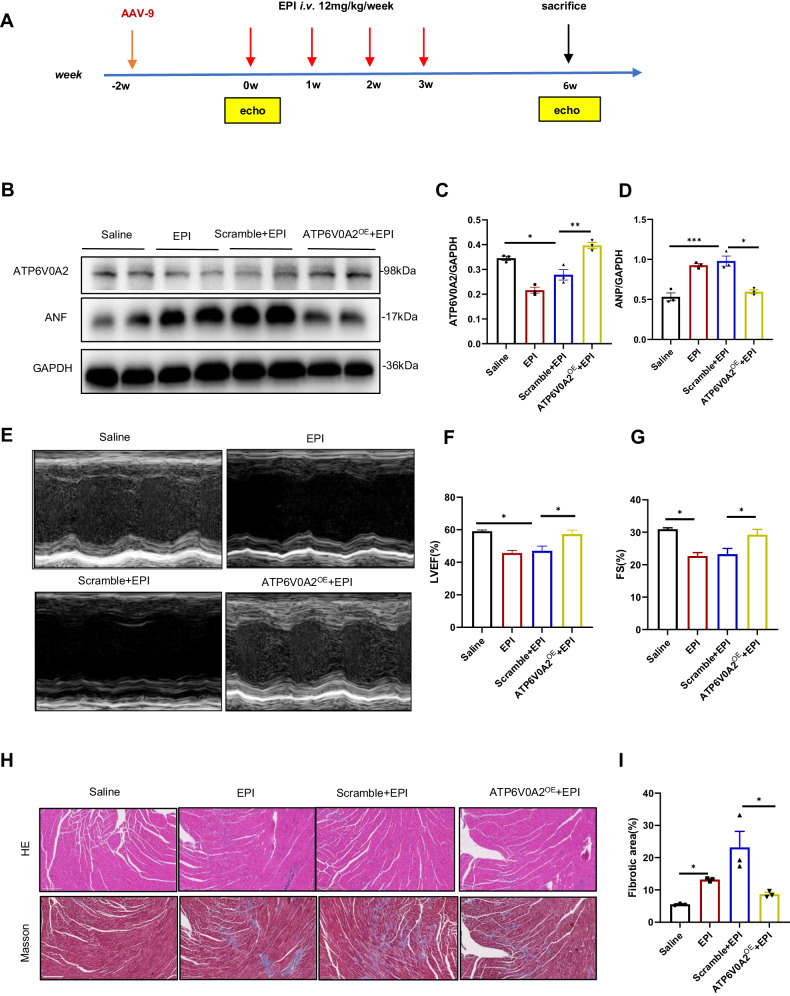
ATP6V0A2 overexpression alleviates EIC in vivo. **A** Experimental design. BALB/C mice in EPI groups, injected with AAV-9 in the indicated groups two weeks before, were exposed to a total dose of 48 mg/kg EPI in next 4 weeks. Mice were detected with echocardiography at the end of the 6th weeks and then sacrificed. **B**–**D** Immunoblotting images of ATP6V0A2 and ANP protein of murine hearts in the indicated groups. **E** Representative left ventricular M-mode echocardiographic tracings. Images are representative of 10 independent mice. **F** Quantitative analysis of LVEF by echocardiography. *n* = 10 mice per group. **G** Quantitative analysis of FS by echocardiography. *n* = 10 mice per group. **H** Representative HE staining images, *n* = 3 mice per group. Scale bar, 50 μm. **I** The quantitative analysis of cardiac sections stained with Masson. One-way ANOVA followed by the Tukey post hoc test was used to compare indicated mice with saline-treated mice. ***p < 0.001, **p < 0.01, *p < 0.05.

To determine the role of ATP6V0A2 in EIC in vivo, AAV-9 carrying cardiomyocyte-specific ATP6V0A2 was applied in mice two weeks before the establishment of EIC models. Then, a total dose of 48 mg/kg EPI (based on our unpublished data, this dosage is comparable to the commonly used 20 mg/kg observed in chronic Dox-induced cardiotoxicity mouse models and it is approximately equivalent to the standard DOX treatment dose of 60 mg/m^2^ administrated to patients [19, 20].) was administrated via tail injection within 4 weeks to establish mouse models of EIC, with echocardiography performed at baseline and 6 weeks after the first EPI administration (Fig. 3A). Following AAV9-ATP6V0A2 treatment, ATP6V0A2 levels in EPI-exposed mouse hearts significantly increased, as measured by IHC staining and immunoblotting (Figs. 1E and 3B, C). We then assessed cardiac function and the extent of myocardial injury of the murine EIC model. Echocardiography analysis revealed that EPI treatment reduced cardiac function in murine hearts, which was significantly improved in hearts overexpressing ATP6V0A2, as quantified by left ventricular EF and FS (Fig. 3E–G). In addition, the fibrotic state of murine hearts was investigated by HE staining and Masson staining. EPI administration resulted in severe cardiac fibrosis, which was mitigated by ATP6V0A2 overexpression (Fig. 3H, I). These pathological changes in EPI-treated murine hearts were accompanied by increased ANP protein levels, a classical biomarker of cardiac hypertrophy. Interestingly, ATP6V0A2 overexpression partially reversed the high ANP expression (Fig. 3B, D).

In summary, these results indicated that cardiac-specific overexpression of ATP6V0A2 protected cardiac function from EPI-induced injury in vivo.

### Ferroptosis is triggered in epirubicin induced cardiotoxicity

**Fig. 4:**
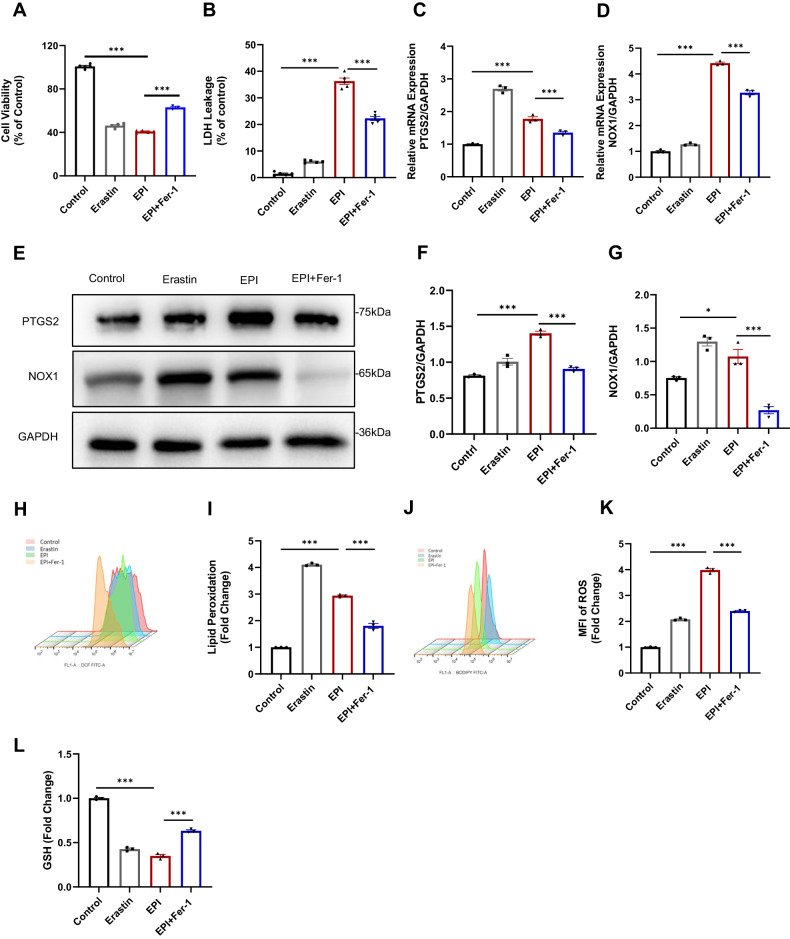
Ferroptosis is triggered in epirubicin induced cardiotoxicity. HL-1 cardiomyocytes were treated with DMSO, erastin(10 μM), EPI(2 μM) or Fer-1 (50 μM) for 12 h. Fer-1 was pretreated for 2 h before the establishment of EIC. **A** Cell viability was measured by CCK-8 of HL-1 cardiomyocytes treated as indicated. **B** LDH release in the medium of HL-1 cardiomyocytes was detected in the indicated groups. The mRNA levels of PTGS2 (**C**) and NOX1 (**D**) in HL-1 cardiomyocytes in the indicated groups. **E**–**G** Immunoblotting images of PTGS2 and NOX1 proteins of HL-1 cardiomyocytes in the indicated groups. **H**, **I** DCFH-DA probe was used to detect the relative ROS level of HL-1 cardiomyocytes in the indicated groups flow cytometry and the quantitative analysis of DCF (FL1) fluorescence are shown. **J**, **K** BODIPY^581/591^- C11 probe was used to detected lipid peroxidation level in HL-1 cardiomyocytes treated with indicated chemicals by flow cytometry and the quantitative analysis of BODIPY^581/591^-C11 (FL1) fluorescence are shown. **L** The relative GSH level of HL-1cardiomyocytes was detected in the indicated groups. One-way ANOVA followed by the Tukey post hoc test was used to compare indicated mice with saline-treated mice. ***p < 0.001, *p < 0.05.

Dexrazoxane, an iron chelator, is currently the only drug used for preventing AIC in cancer patients, providing early evidence to the relationship between cardiotoxicity and iron. Ferroptosis, a novel regulated cell death characterized by iron-dependent lipid peroxidation, underscores the critical role that iron plays in the procession [16]. Previous publications have indicated that ferroptosis participated in the progression of DIC [21, 22]. Therefore, it is conceivable that ferroptosis is also triggered in EIC. To investigate whether ferroptosis is involved in EIC, we used erastin, a ferroptosis activator, on HL-1 cardiomyocytes as a positive control of ferroptosis. Our analysis revealed that erastin and EPI led to a considerable decline in cell viability and a sharp increase of LDH release. These effects were attenuated by Fer-1, a ferroptosis inhibitor, suggesting that ferroptosis is involved in EIC (Fig. 4A, B).

Next, we investigated changes in two markers related to ferroptosis, PTGS2 and NOX1. Our studies showed that the expression of PTGS2 and NOX1 at both mRNA and protein levels increased following treatment with erastin or EPI. However, these increases were reversed by co-treatment with Fer-1 (Fig. 4C–G). Additionally, we detected intracellular ROS and lipid-peroxidation, two key markers of ferroptosis. DCFH-DA staining demonstrated an overwhelming generation of ROS in EPI-treated cardiomyocytes (Fig. 4H, I), and a similar trend of lipid-peroxidation was indicated by BODIPY^581/591^-C11staining (Fig. 4J, K). In addition, the content of GSH, a marker of oxidative stress, showed a significant decrease in HL-1 cardiomyocytes treated with erastin and EPI, suggesting elevated lipid peroxidation level induced by EPI (Fig. 4L). Notably, these aberrant alterations in EPI-treated cardiomyocytes were all attenuated Fer-1. Overall, these findings are consistent with the notion that ferroptosis was activated in EIC.

### Forced expression of ATP6V0A2 mitigates ferroptosis in cardiomyocytes and murine hearts

**Fig. 5:**
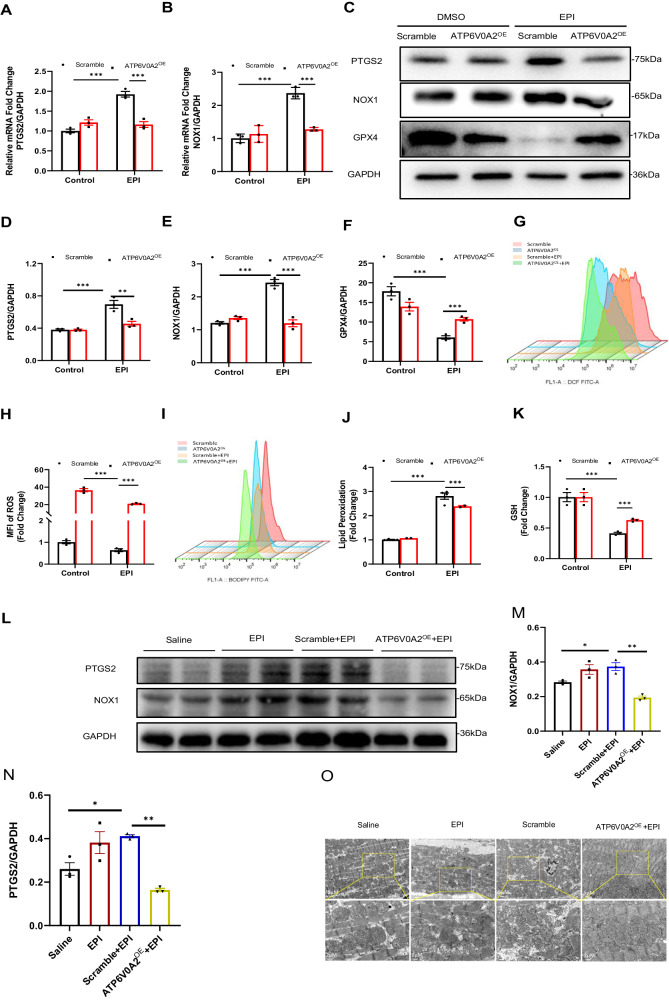
Forced expression of ATP6V0A2 mitigates ferroptosis in cardiomyocytes and murine hearts. HL-1 cardiomyocytes were transfected with plasmids of scramble or ATP6V0A2 in advance, and then treated with DMSO or EPI (2 μM) for 12 h. The mRNA levels of PTGS2 (**A**) and NOX1 (**B**) in HL-1 cardiomyocytes in the indicated groups. **C**–**F** Immunoblotting analysis of PTGS2, NOX1 and GPX4 proteins of HL-1 cardiomyocytes in the indicated groups. **G**, **H** DCFH-DA probe was used to detect the relative ROS level of HL-1cardiomyocytes in the indicated groups flow cytometry and the quantitative analysis of DCF (FL1) fluorescence are shown. **I**, **J** BODIPY^581/591^-C11 probe was used to detected lipid peroxidation level in HL-1 cardiomyocytes treated with indicated chemicals by flow cytometry and the quantitative analysis of BODIPY^581/591^ -C11 (FL1) fluorescence are shown. **K** The relative GSH level of HL-1 cardiomyocytes was detected in the indicated groups. **L**–**N** The protein abundance of PTGS2 and NOX1 in murine hearts in the indicated groups. *n* = 3 mice per group. **O** Representative images of transmission electron microscopy show morphology of the mitochondrion. Scale bars are shown in the images. One-way ANOVA followed by the Tukey post hoc test was used to compare indicated treatment groups and control group in murine hearts. Two-way ANOVA and subsequent Tukey test was performed to compare groups with different secondary treatments in HL-1 cells. ***p < 0.001, **p < 0.01, *p < 0.05.

To elucidate the role of ATP6V0A2 in ferroptosis, ATP6V0A2 overexpression plasmids were transfected into the cardiomyocytes. In our study, EPI induced changes in the transcript and protein levels of GPX4, PTGS2 and NOX1 were markedly reversed by ATP6V0A2 overexpression in cardiomyocytes (Fig. 5A–F). In addition, after exposure to EPI, cardiomyocytes with ATP6V0A2 overexpression exhibited reduced levels of ROS and lipid peroxidation (Fig. 5G–J). Similar changes were detected in EPI-treated murine hearts with ATP6V0A2 overexpression (Fig. 5L–N), suggesting that ATP6V0A2 was involved in the process of ferroptosis. Meantime, we found ATP6V0A2 overexpression increased the content of GSH, which was decreased in cardiomyocytes treated with EPI (Fig. 5K), indicating that ATP6V0A2 alleviates the massive production of lipid-peroxidation. EPI-treated murine cardiomyocytes had smaller mitochondria, increased membrane densities and signs of cell fragmentation, which was the typical ferroptosis related morphological features under transmission electron microscopy [23]. Interestingly, ATP6V0A2 overexpression rescued ferroptosis related mitochondrial injuries in murine hearts (Fig. 5O). Collectively, these findings propose that ATP6V0A2 inhibits ferroptosis in EIC both in vitro and in vivo.

### Forced expression of ATP6V0A2 reacidify the lysosome compartment of cardiomyocytes against EIC

It is known that lysosomes are reservoirs of iron in the cell and the damage in lysosomes can cause the release of lysosomal iron and subsequent production of ROS [11], which may induce ferroptosis [14]. Another report has shown that doxorubicin inhibits V-ATPase–driven lysosome acidification [13]. Therefore, we examined whether EPI alters lysosomal acidification in HL-1 cardiomyocytes.

**Fig. 6:**
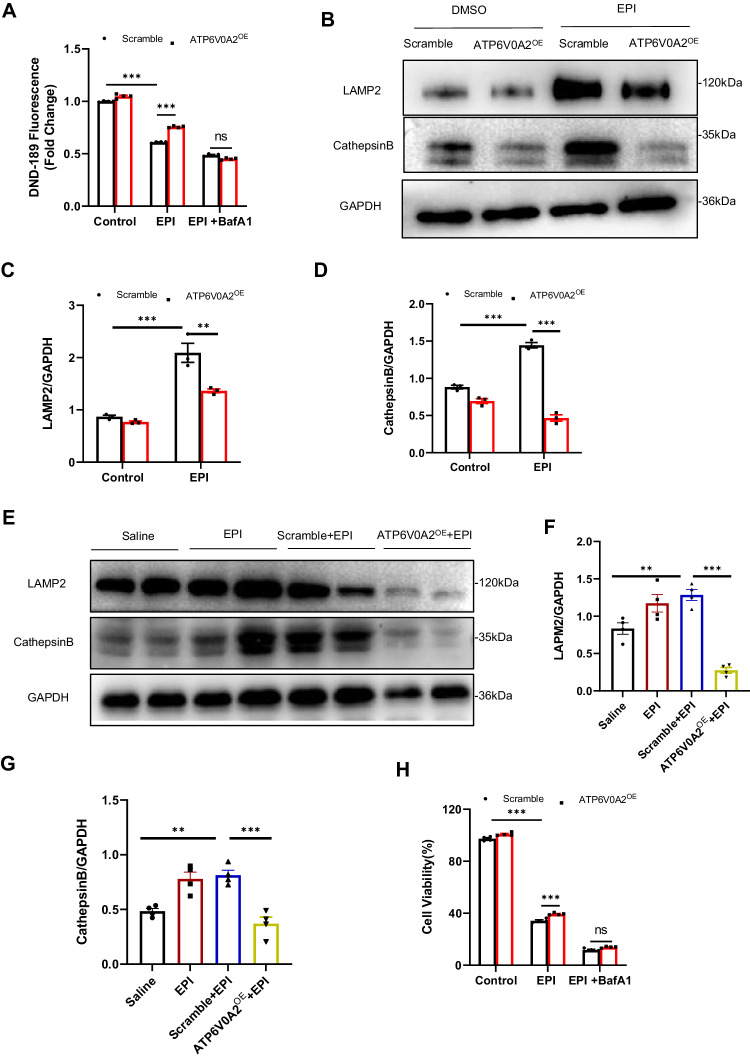
Forced expression of ATP6V0A2 reacidify the lysosome compartment of cardiomyocytes against EIC. **A** Relative quantification of LysoSensor DND-189 fluorescence of HL-1 cardiomyocytes in the presence or absence of EPI (2 μM) or BafA1(50 nM) was measured by flow cytometry. **B**–**D** Immunoblotting images of lysosomal function related proteins (LAMP2 and Cathepsin B) in HL-1 cardiomyocytes are shown. **E**–**G** Immunoblotting images of LAMP2 and Cathepsin B proteins in murine hearts in the indicated groups are shown. *n* = 3 mice per group. **H** Cell viability of HL-1 cardiomyocytes in the indicated groups was detected by CCK-8. One-way ANOVA followed by the Tukey post hoc test was used to compare indicated treatment groups and control group in murine hearts. Two-way ANOVA and subsequent Tukey test was performed to compare groups with different secondary treatments in HL-1 cells. ***p < 0.001, **p < 0.01, ns indicates no sense.

Firstly, we measured lysosomal pH qualitatively using LysoSensor DND-189. DND-189 emits green fluorescence, the intensity of which increases within an acidic environment. The data showed that EPI decreased DND-189 fluorescence, suggesting that EPI decreases lysosomal acidity in cardiomyocytes. Interestingly, the elevated lysosomal luminal pH after EPI exposure was significantly restored by overexpression of ATP6V0A2. However, when co-treatment with BafA1, a well-established V-ATPase inhibitor, the acidic environment restored by overexpression of ATP6V0A2 again alkalized within the lysosome compartment, suggesting that ATP6V0A2 -dependent lysosome acidification plays a vital role in EIC (Fig. 6A). Additionally, we examined the expression of two lysosome-specific markers, LAMP 2 and Cathepsin B, to investigate the effect EPI exposure on the lysosomal dysfunction. Our data demonstrated that EPI increased the abundance of LAMP2 and Cathepsin B in vivo and in vitro, which were decreased with overexpression of ATP6V0A2 (Fig. 6B–D). These results further verified that ATP6V0A2 protected lysosomal function from EIC, consistent with the in vivo data (Fig. 6E–G).

To explore the role of ATP6V0A2 in ferroptosis triggered in EIC, we examined the effect of BafA1 on EPI treated cardiomyocytes. Interestingly, we observed that the protection effect of ATP6V0A2 overexpression against EIC was abolished by BafA1 in cardiomyocytes (Fig. 6H). Together, these data revealed that EPI impairs lysosomal acidification driven by ATP6V0A2, and inhibition of this process induces ferroptosis and promotes EIC.

## Discussion

**Fig. 7:**
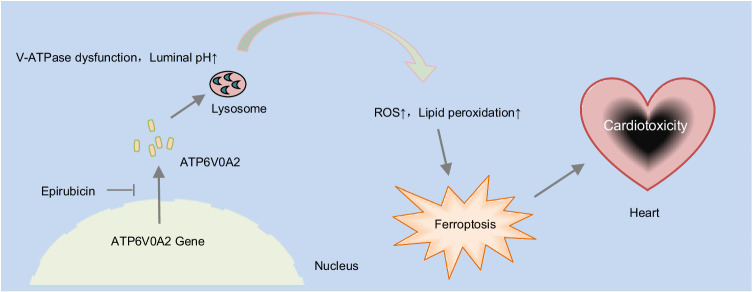
Working model. Epirubicin, inhibiting lysosomal acidification and lysosomal function via decreasing ATP6V0A2 expression, leads to increased ROS production and lipid peroxidation, motivating cardiomyocyte ferroptosis and induces cardiac injury. Maintenance of lysosomal acidification by increasing ATP6V0A2 partially blunts ferroptosis and protects heart from eEIC.

In this study, we demonstrate that EPI impairs ATP6V0A2-drived lysosomal acidification and function, thereby inhibiting ferroptosis in cardiomyocytes. This impairment, in turn, is associated with the development of cardiotoxicity. We also provide evidence that increased expression of ATP6V0A2 in cardiomyocytes ameliorates EIC. Collectively, these findings reveal the mechanism that ferroptosis is inhibited by ATP6V0A2-drived lysosomal acidification in EIC and highlight a potential target for improving EIC (Fig. 7).

Due to its dose-dependent cardiotoxicity, the clinical application of EPI is limited, often affecting patients’ quality of life. However, the exact mechanism by which EPI exerts its toxic effects on cardiomyocytes remains elusive. In this regard, we performed cDNA microarray analysis and found that ATP6V0A2 was significantly decreased in EPI-treated cardiomyocytes. To confirm this finding, we further investigated the changes of ATP6V0A2 at both transcript and protein levels in vivo and in vitro, strongly supporting the conclusion that EPI induced a reduction in ATP6V0A2. However, it has been reported that the protein level of ATP6V0A2 in cardiomyocytes manifested no changes after doxorubicin treatment [19]. We believe the discrepancy may be due to differences in drug type and its interference conditions. ATP6V0A2 is a subunit of the V0 complex of V-ATPases, mutations of which in the Golgi-targeted human V-ATPase results in autosomal recessive cutis laxa type II, a disorder characterized by defects in glycosylation and membrane trafficking [24]. Recently, ATP6V0A2 is reported to be implicated in ovarian cancer [25] and breast tumor [26]. Beaman and his colleagues have published serial studies demonstrating that ATP6V0A2-drived apoptosis modulates of fertilization in male and female [27–29]. These findings underscore the key role that ATP6V0A2 plays in a variety of diseases. However, the role of ATP6V0A2 in EIC has not been investigated. Herein, our data suggest that the overexpression of ATP6V0A2 significantly alleviates cardiotoxicity induced by EPI in HL-1 cardiomyocytes and murine hearts. It is the first time that the important role of ATP6V0A2 in EIC was identified, providing a novel target for EIC alleviation.

DXZ, as an iron chelator, is widely used in clinical practice to prevent cardiotoxicity. And iron, a dependent factor in ferroptosis, plays a pathogenic role in cardiotoxicity in several cardiopathic conditions [15, 16, 30]. In this regard, it is conceivable to link ferroptosis to cardiomyocyte damage. Ferroptosis is a novel regulated cell death, characterized by iron dependence [8] and following membrane lipid peroxidation. Discovered in the procession of screening new chemical small molecule drugs to trigger the death of RAS mutation cancer cells, it has been validated that ferroptosis participated in various pathological conditions, including chemotherapy-induced cancer cell death [31], acute kidney injury [32], and neurodegenerative diseases [33]. In fact, some literature has reported the vital role of ferroptosis in cardiotoxicity induced by doxorubicin. In 2019, Fang et al. [7] initially reported that inhibition of ferroptosis, rather than autophagy or other kinds of cell death, could significantly alleviate cardiotoxicity and increase mouse survival after doxorubicin treatment, indicating that ferroptosis mainly contributes to the progression of DIC. Another study showed that GPX4-transgenic mice after doxorubicin exposure improve cardiac performance by ferroptosis inhibition [17]. Consistent with these early studies, our pathway analysis of microarray data suggested the involvement of ferroptosis in EIC. To further confirm the role of ferroptosis in EIC, we conducted a comprehensive evaluation of ferroptosis both in vivo and in vitro. Our results showed that EPI treatment led to significant changes in ferroptosis-related markers, including COX2 and NOX1, mitochondrial damage and lipid-ROS. Interestingly, these aberrant parameters induced by EPI were significantly attenuated in cardiomyocytes overexpressing ATP6V0A2. Taken together, we conclude that ferroptosis plays an important role in EIC, and ATP6V0A2 protects cardiomyocytes against EIC by inhibiting ferroptosis.

Maintenance of cellular homeostasis relies on degradation functions mediated by lysosomal functions. The lysosomal degradation depends on the proper acidic environment, which is crucial to the lumen-residing acid hydrolases. The lysosomal pH can be altered by the expression level of V-ATPase at membranes of lysosomes, the rate of proton leak across the organellar membrane and the degree of V-ATPase assembly. Recent report have indicated that lysosomal acidification accounts for doxorubicin induced cardiac injury [19]. Consistent with this, we observed a significant increase in lysosomal pH in EPI-treated cardiomyocytes. ATP6V0A2 is a2 subunit of the V-ATPase, and mutations in this subunit are associated with cutis laxa, characterized by loose and inelastic skin and by abnormalities in other connective tissues including the blood vessels and joints [24]. However, the role of ATP6V0A2 in EIC has not been investigated. In our study, overexpression of ATP6V0A2 largely rescued the increased lysosomal pH in cardiomyocytes, suggesting a pivotal role of ATP6V0A2 in EPI induced lysosomal dysfunction.

However, we observed an interesting phenomenon: overexpression of ATP6V0A2 did not enhance lysosomal acidity in cardiomyocytes that were not treated with EPI. A recent study showed that TMEM175, a proton-activated, proton-selective channel on the lysosomal membrane, is activated to terminate lysosomal acidification beyond the normal range by mediating the lysosomal H^+^ leak [34]. This explained the stable lysosomal acidity in cardiomyocytes overexpressing ATP6V0A2 without EPI treatment. The lysosomes act as one of the main iron storage sites. Damage to lysosomes causes the leakage of lysosomal iron and subsequent production of ROS by Fenton reaction, a good ignitor of ferroptosis [18]. Moreover, increased ROS exacerbates lysosomal injury, further promoting ferroptosis [35]. On the other hand, mTOR-TFEB pathway is reported to inhibit ferroptosis by lysosomal stress [36]. Taken together, ATP6V0A2 might inhibit ferroptosis in EIC by modulating lysosomal acidity. Our data showed that ATP6V0A2 fails to restore the lysosomal acidity and improve cell viability on EPI exposure when co-treated with BafA1, an inhibitor of V-ATPase. This indicates that ATP6V0A2 restores lysosomal luminal acidity via V-ATPases, suggesting that ATP6V0A2 enhances the activity of V-ATPases.

Additionally, we found ATP6V0A2 overexpression significantly suppresses ferroptosis in EIC, which is blunted by BafA1. Based on the current findings, we proposed a model in which ATP6V0A2 mitigates ferroptosis triggered by EPI-exposure by maintaining lysosomal acidification.

In summary, we initially demonstrated the novel role of ATP6V0A2 in restoring lysosomal acidification to inhibit ferroptosis in EIC. Our improving understanding of the role ATP6V0A2 plays in ferroptosis may provide a new therapeutic target for EIC.

## Material and methods

### Cell culture

HL-1 cell line was purchased from Procell and tested for no mycoplasma contamination. Cells were cultured in Modified Eagle Medium (MEM) containing 10% fetal bovine serum (FBS, Sigma) at 37 °C in a humidified 5% CO_2_-jacked incubator. Upon the confluence of cells reaching 50–60%, HL-1 cardiomyocytes were respectively treated with dimethyl sulfoxide (DMSO), erastin (10 μM, MedChemExpress), epirubicin (EPI, 2 μM, MedChemExpress) with or without Ferrostatin-1 (Fer-1, 50 μM, Sigma) for 12 h. To further identify the role of lysosomal acidification in EIC, Bafilomycin A1 (BafA1, 50 nM, MedChemExpress) was used to inhibit V-ATPases for 12 h. For overexpression of ATP6V0A2 in vitro, plasmids of ATP6V0A2 and scramble (GeneChem) were transfected into HL-1 cardiomyocytes using Lipofectamine^TM^ 3000 transfection reagent (Invitrogen). Then the transfected cardiomyocytes were used in the subsequent experiments.

Neonatal mouse ventricular myocytes (NMVMs) were isolated with Pierce Primary Cardiomyocyte Isolation Kit (Thermo Fisher Scientific) according to the manufacture’s instruction. Briefly, fresh 3-day neonatal mice hearts were placed into sterile micro-centrifuge tubes and cold Hanks’ Balanced Salt Solution (HBSS) was added for washing immediately. Then, each heart was minced into 1–3 mm^3^ pieces and incubated with indicated mixed enzyme in a 37 °C incubator for 30 min. After that, Dulbecco’s modified Eagle’s medium (DMEM, Gibco) supplemented with 10% FBS was used for preparing single-cell suspension by pipetting up and down. After incubation for 2 h, fibroblasts were cleared so that cardiomyocytes were collected and then plated in DMEM containing 10% FBS and bromodeoxyuridine (BrdU, 100 μM, MedChemExpress). After being cultured for 48 h, NMVMs were used for experiments.

### cDNA microarray

Total RNA was extracted from NMVMs treated with 2 μM EPI for 24 h and purified with RNeasy mini kit (QIAGEN) according to the manufacturer’s instructions. The microarray analysis was performed using Affymetrix GeneChip probe arrays (Thermo Fisher Scientific). *P*-values were adjusted with a false discovery rate of 5%. Controls were implemented at each step to ensure data quality.

### Cell viability assay

Cell viability assay was performed with the Cell Counting Kit (CCK-8, Sigma-Aldrich) according to the manufacturer’s instructions. Briefly, HL-1 cardiomyocytes were seeded at 10^4^ cells per well in a 96-well plate. After the indicated treatment, cells were incubated with CCK-8 at 37 °C for 2 h and then the absorbance signal was measured at 450 nm using a microplate reader (BioTek). Based on the recorded absorbance, the percentage of cell viability was then calculated accordingly.

### Lactate dehydrogenase (LDH) release assay

HL-1 cardiomyocytes were inoculated at the density of 10^4^ cells per well in a 96-well plate. After the indicated treatment, culture supernatant was collected. And an LDH release assay kit (Beyotime) was used following the manufacturer’s instructions to assess the degree of LDH release. Absorbance at 495 nm was read by a microplate reader (BioTek).

### Quantitative real-time PCR (qRT-PCR)

Total RNA was extracted from HL-1 cardiomyocytes using TRIzol (Vazyme), and the concentration and purity of RNA were measured using spectrophotometry. A total of 1 μg RNA was reversed for transcription using HiScript III RT SuperMix for qPCR (+gDNA wiper) (Vazyme) in accordance with the manufacturer’s instructions. Subsequent qPCR was performed using ChamQ Universal SYBR qPCR Master Mix (Vazyme) according to the manufacturer’s instructions. Presented relative to GAPDH mRNA, the fold change in gene expression was calculated using the 2^−△△Ct^ method.

Primer sequences are below:

- *Atp6v0a2, 5‘-GTGCAGTTCCGAGACCTCAA-3‘(forward);*
  *5‘-GTTTAAGAGGTGGTGCGGGA-3‘(reverse)*.
- *Ptgs2, 5‘- CCTGAGCGGTTACCACTTCA-3‘ (forward);*
  *5‘- TGAGTGAGTCCATGTTCCAGG-3‘(reverse)*.
- *Nox1, 5‘ -TCACGACCTGCCAAAGAGAC-3‘ (forward);*
  *5‘-CCACGAAACAAAGCCAAGCA-3‘(reverse)*.
- *Gapdh, 5‘-ATCATCCCTGCATCCACT-3‘ (forward);*
  *5‘-ATCCACGACGGACACATT-3‘(reverse)*.

### Immunoblotting analysis

Total protein was extracted from ventricular tissues or HL-1cardiomyocytes by homogenizing in RIPA buffer (Epizyme) containing protease inhibitors (MedChemExpress). The homogenate was cleared by centrifugation at 4 °C for 30 min at 12,000 rpm to collect protein in the supernatant. Protein concentration in the supernatant was measured using the BCA Protein Quantification Kit (Vazyme). Protein lysates of 20 μg were loaded per sample for electrophoresis and transferred to a Polyvinylidene Fluoride (PVDF) membrane. Then, the membranes were blocked with 5% skimmed milk and incubated with primary antibodies at 4 °C overnight. The following antibodies were used: anti-NOX1 (1:800, Proteintech, #17772-1-AP); anti-COX2 (1:1000, Abmart, #T58852S); anti-ATP6V0A2 (1:1000, Abcam, #ab96803); anti-GAPDH (1:5000, Proteintech, #60004-1-Ig). Washed sufficiently, the membranes were then probed with the appropriate species of horseradish peroxidase‒conjugated secondary antibodies (Proteintech, #SA00001-1, #SA00001-2) and detected with the Pierce ECL System (Thermo Fisher Scientific).

### ROS production assay

HL-1 cardiomyocytes were seeded at a density of 5$\times$10^5^ per well in a 6-well plate. The cells were incubated with DCFH-DA in a 37 °C incubator in the dark for 30 min after 12 h of treatment as indicated, according to the manufacturer of Oxygen Species Assay Kit (Beyotime). And then single-cell suspension was prepared and 10^4^ cells were detected with the flow cytometer (Beckman Coulter) in the FL-1 channel. Data were analyzed by FlowJo. software.

### Lipid-peroxidation assay

HL-1 cardiomyocytes were seeded at a density of 5$\times$10^5^ per well in a 6-well plate and treated as indicated. After 12 h of treatment, the cardiomyocytes were incubated with 2 µM BODIPY^581/591^-C11(Invitrogen) for 30 min at 37 °C in the dark. Then the BODIPY^581/591^-C11 emission was detected using the FL-1 channel on a flow cytometer (Beckman Coulter). Data collected from 10^4^ cells was analyzed by FlowJo. software.

### Lysosomal pH measurement

For qualitative measurement of lysosomal pH, cardiomyocytes were loaded with 2 μM Lysosensor Green DND-189 (Thermo Fisher Scientific) at 37 °C for 30 min and then washed with PBS for 3 times and prepared into single-cell suspension. Each sample with 10^4^ cardiomyocytes were detected in the FL-1 channel for flow cytometry and analyzed by FlowJo. software.

### Animal models

A total of 40 BALB/C male mice aged 6–8 weeks were maintained on a 12-h light/dark cycle from 6 AM to 6 PM. The mouse experimental protocols used in this study were approved by the Ethics Committee of Nanjing Medical University (IACUC-1910015). After adaptive feeding for 3 days, the experiment was then performed. An adeno-associated virus serotype 9 (AAV9)-based delivery system combined with a cardiac troponin T promoter (cTnT) was used for forced expression of cardiomyocyte-specific ATP6V0A2. The AAV9 vectors carrying ATP6V0A2 overexpression sequence (AAV9-ATP6V0A2) or scramble (AAV9-Scramble) were designed and constructed by Shanghai GeneChem, and the cTNTp-MCS-3flag-T2A-EGF vector was used for AAV9-ATP6V0A2 and AAV9-scramble packaging.

Male BALB/c mice were blindly randomized into four different treatment groups: Saline, EPI, Scramble + EPI, and ATP6V0A2^OE^ + EPI. For groups of Scramble and ATP6V0A2^OE^ + EPI mice, each mouse was administrated with 0.2 mL (10E + 11 vg) of a solution containing AAV9-ATP6V0A2 or AAV9-scramble by an intravenous tail injection, which was diluted with saline, and housed for 2 weeks before EPI injection. Subsequently, mice were injected via the tail vein with EPI (12 mg/kg) or normal saline once weekly for 4 weeks. Ventricular function was examined by echocardiography 2 weeks after the final injection. All mice were then euthanized, and the hearts were harvested for further experiments.

### Echocardiography

Transthoracic echocardiography was performed on anesthetized mice (Vevo 2100). M-mode images of the left ventricle were obtained at the level of the papillary muscles.

The echocardiography specialist was not informed the specific treatments on the mice, which would only be revealed upon completion of the analysis. Percentage of fractional shortening (FS) and left ventricular ejection fraction (LVEF) were calculated using the above-mentioned primary measurements and the accompanying software. Five representative contraction cycles were selected for analysis.

### Histology

Firstly, murine hearts were fixed with 4% paraformaldehyde and embedded into paraffin. Then, paraffin sections were subjected to 4 mm sequential slices for the next Hematoxylin-Eosin (HE). To measure collagen deposits, select sections were stained Masson. Images were obtained by microscope (Olympus).

### Immunohistochemistry

Serial murine heart sections were deparaffinized, and blocked with phosphate-buffered saline (PBS) containing 5% normal goat serum and 1% BSA. Then the sections were incubated overnight with rabbit anti-mouse-ATP6V0A2 (1:300, Abcam) at 4 °C under humidified conditions, followed by incubation for 1 h with anti-rabbit secondary antibody (1:500, Proteintech) at room temperature. Images were obtained using an Eclipse E400 microscope (Nikon).

### Transmission electron microscopy

Samples of the myocardium (1 mm $\times \,$ mm $\times \,$ mm) were quickly removed from the left ventricle and immediately fixed in 3% phosphate-glutaraldehyde. Then the cardiac tissues underwent permeation, dehydration, and embedding overnight. The samples were viewed using a transmission electron microscope at an accelerating voltage of 200 kV. (HITACHI).

### Data analysis

For each set of experiments, the sample size was chosen to ensure adequate power to detect variations. The data was in a normal distribution, and variance was similar between the groups that are being statistically compared. Data were analyzed with Prism software 9.0. All data are reported as mean ± SEM. The Student’s *t* test (two-tailed) was performed to compare 2 groups. One-way ANOVA followed by the Tukey post hoc test was used to compare multiple groups. Two-way ANOVA and subsequent. Tukey test was performed to compare groups with different secondary treatments. *P*-values were adjusted with a false discovery rate of 5%. A value of *p* < 0.05 was considered significant.

### Supplementary information

Original uncropped western blots

## Data Availability

Data will be made available on request.
