# Supplementary material for: Epirubicin induces cardiotoxicity through disrupting ATP6V0A2-dependent lysosomal acidification and triggering ferroptosis in cardiomyocytes
Source: Cell Death Discov. 2024 Jul 24;10:337. doi: 10.1038/s41420-024-02095-z (PMC11269639; doi:10.1038/s41420-024-02095-z)
Supplement: Supplementary file 1 — Original uncropped western blots [file 41420_2024_2095_MOESM1_ESM.pptx]

## Slide 1
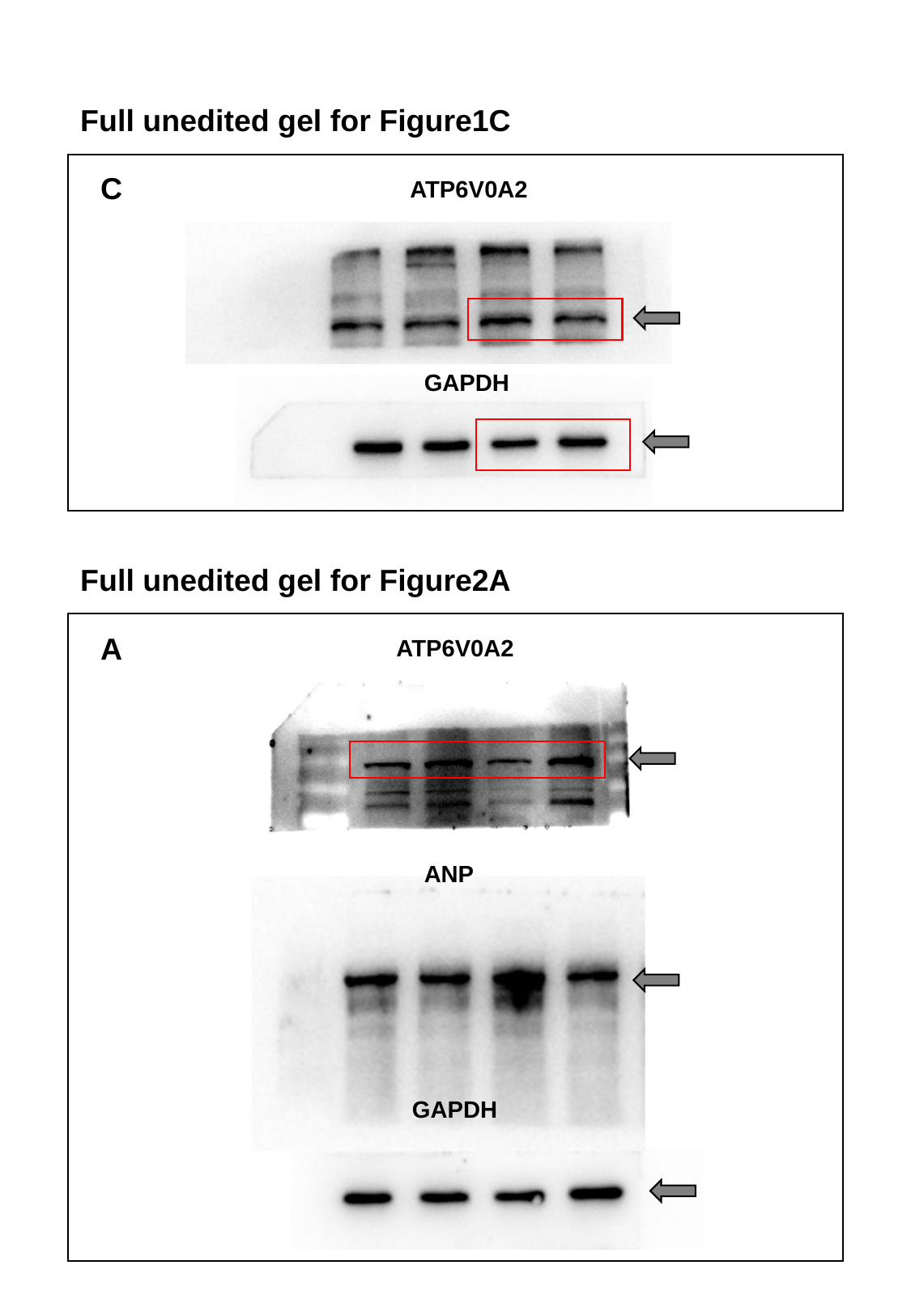

Full unedited gel for Figure1C
C
ATP6V0A2
GAPDH
Full unedited gel for Figure2A
A
ATP6V0A2
ANP
GAPDH

## Slide 2
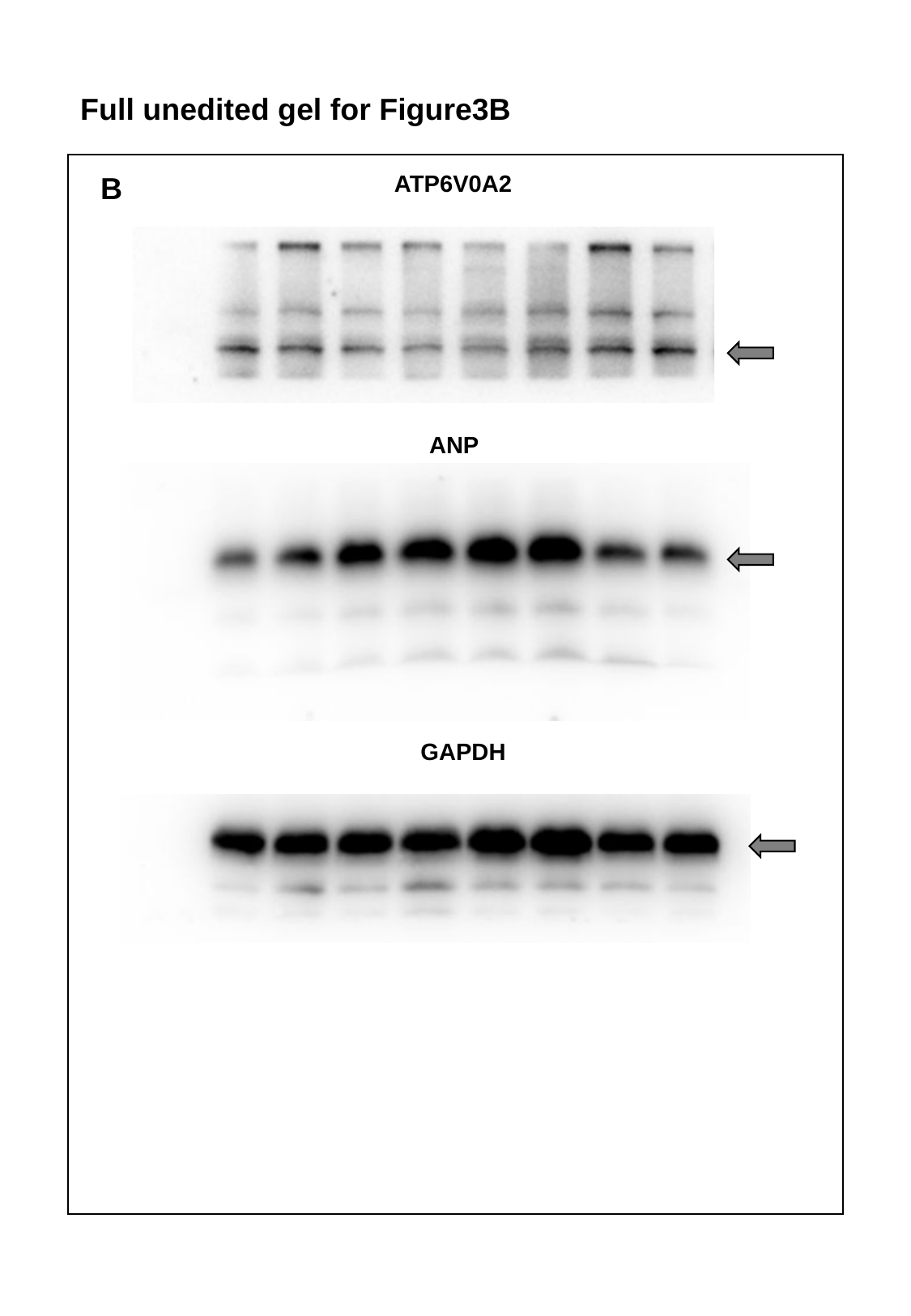

Full unedited gel for Figure3B
B
ATP6V0A2
ANP
GAPDH

## Slide 3
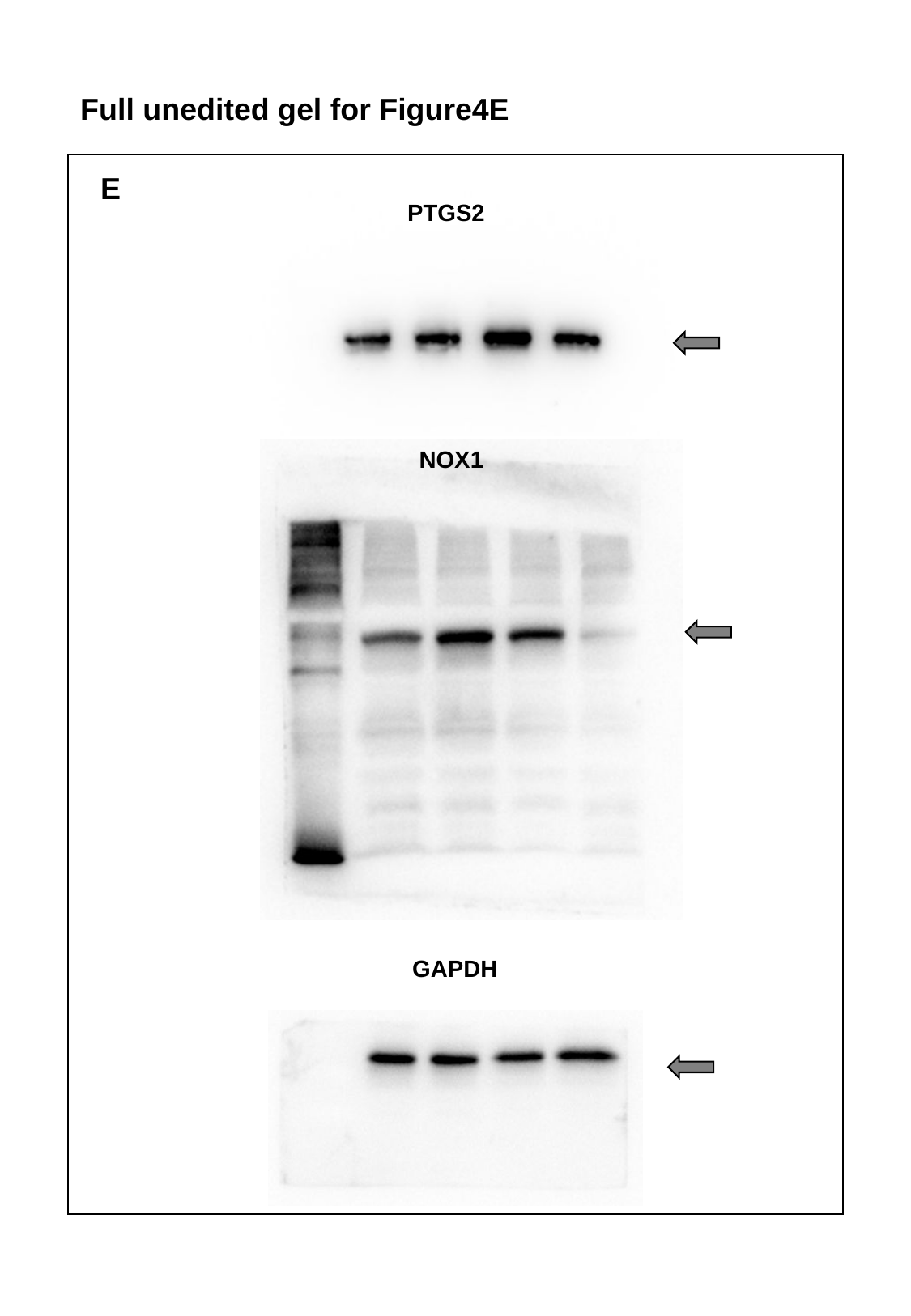

Full unedited gel for Figure4E
E
PTGS2
NOX1
GAPDH

## Slide 4
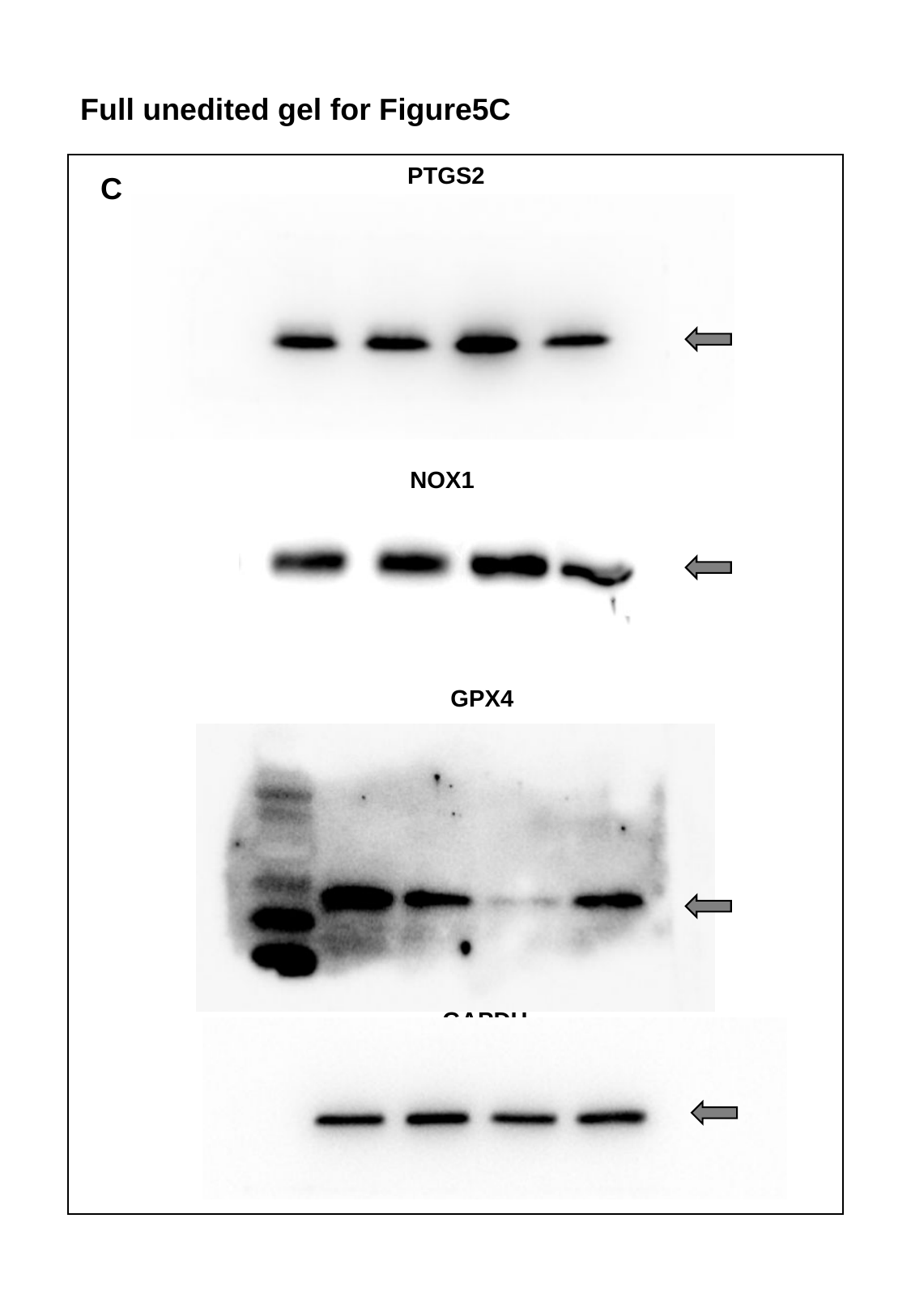

Full unedited gel for Figure5C
PTGS2
C
NOX1
GPX4
GAPDH

## Slide 5
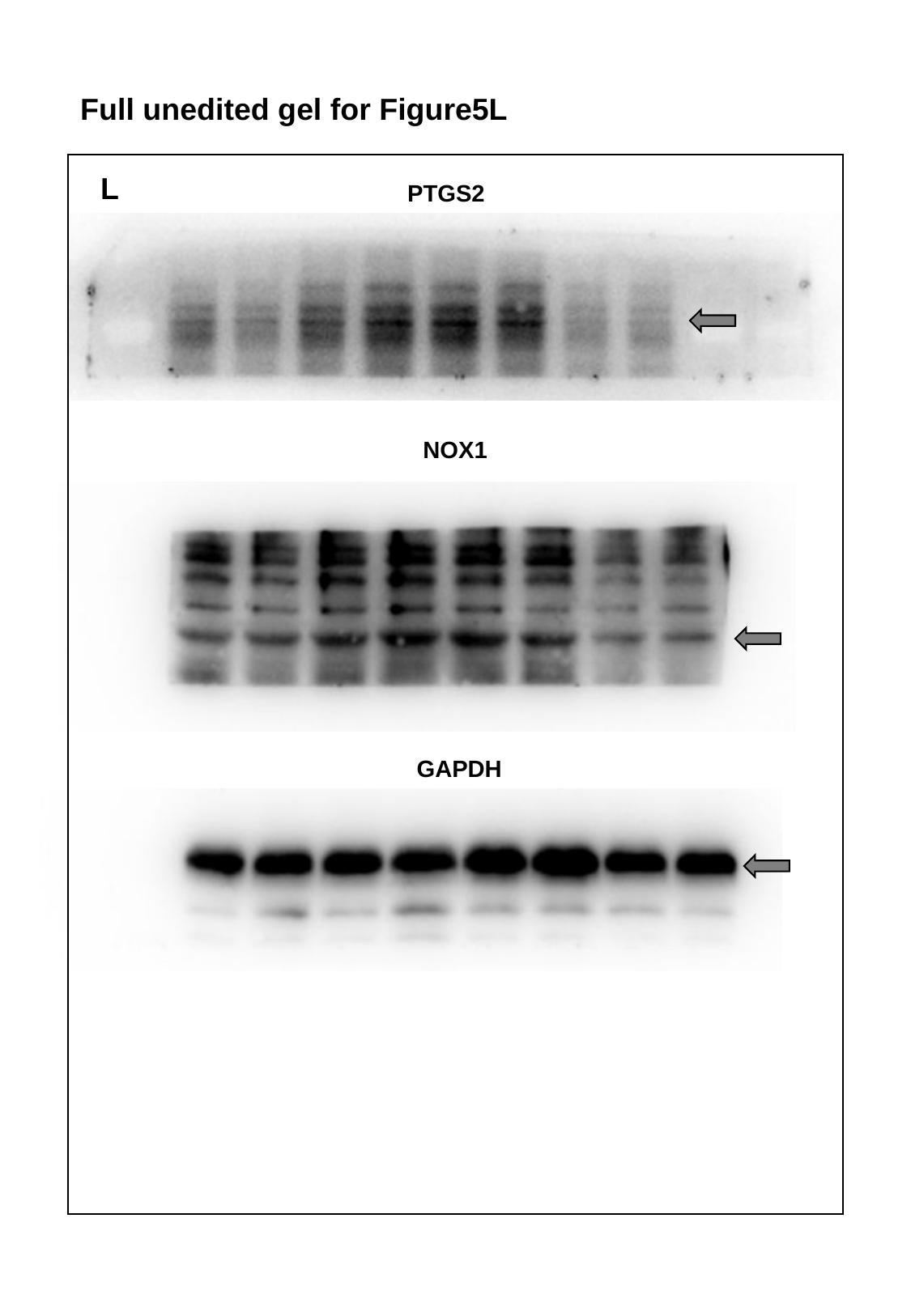

Full unedited gel for Figure5L
L
PTGS2
NOX1
GAPDH

## Slide 6
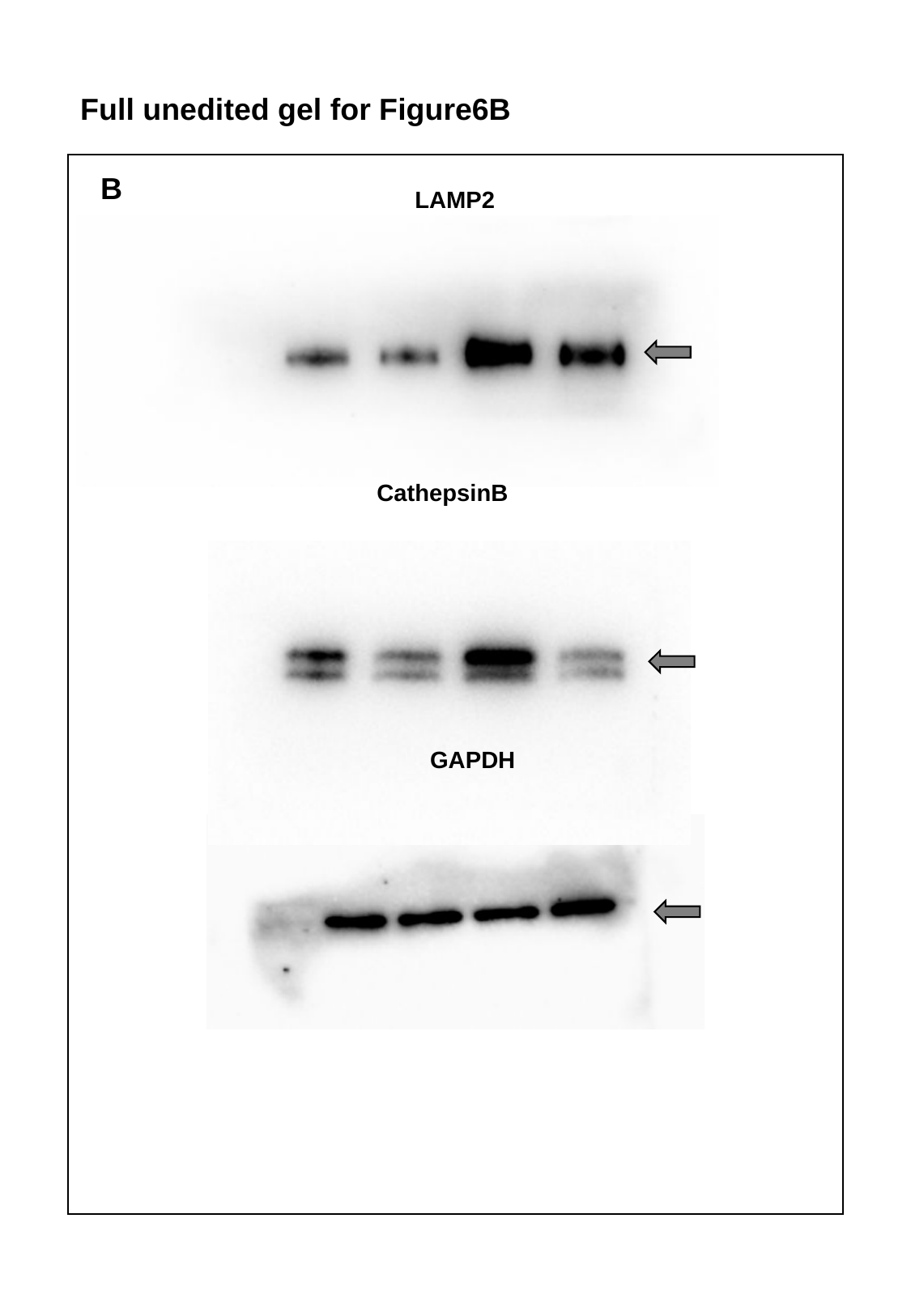

Full unedited gel for Figure6B
B
LAMP2
CathepsinB
GAPDH

## Slide 7
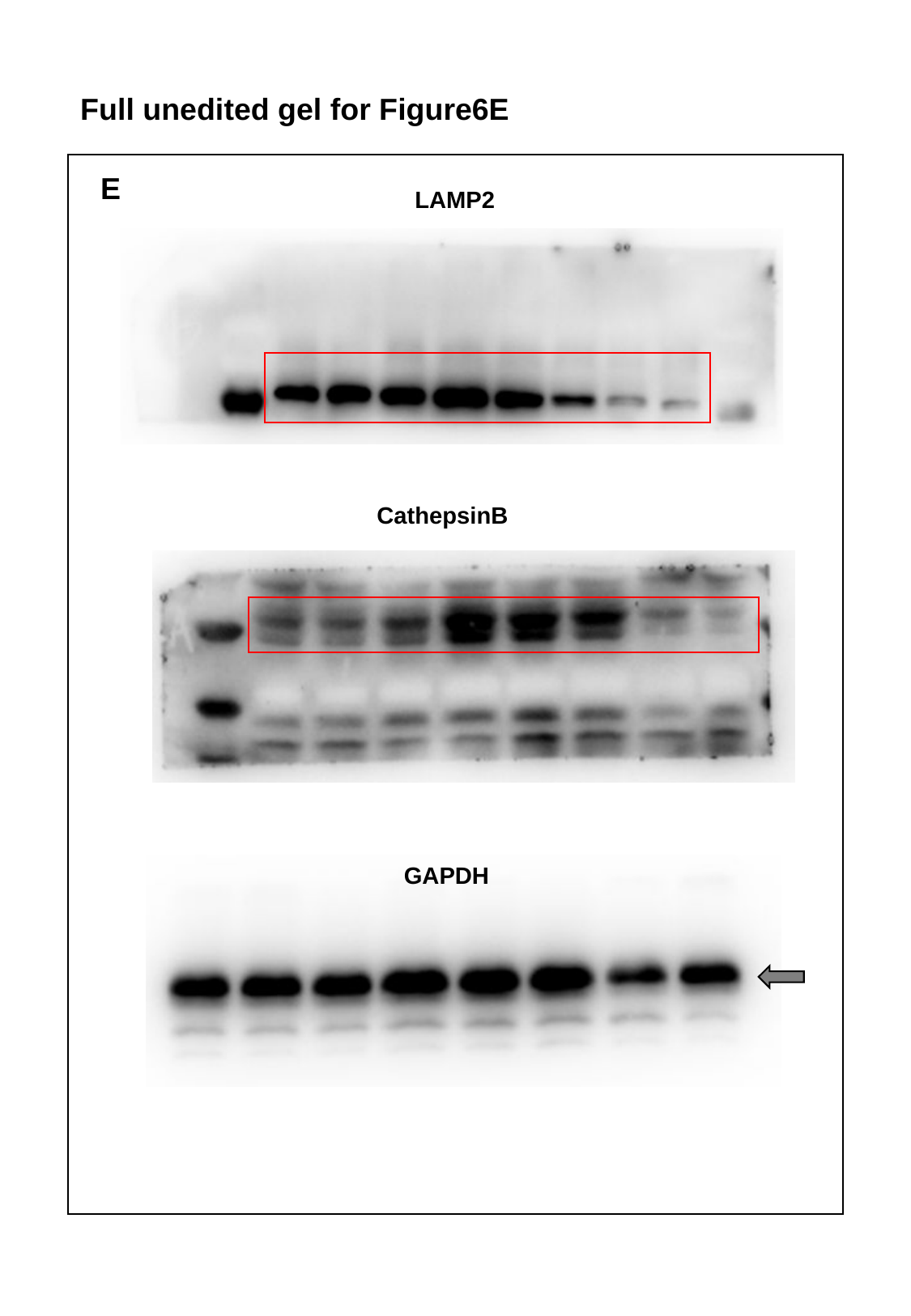

Full unedited gel for Figure6E
E
LAMP2
CathepsinB
GAPDH
